# Multiple Confidence Intervals and Surprisal Intervals to Avoid Significance Fallacy

**DOI:** 10.7759/cureus.51964

**Published:** 2024-01-09

**Authors:** Alessandro Rovetta

**Affiliations:** 1 Research and Disclosure Division, R&C Research, Bovezzo (BS), ITA; 2 Technological and Scientific Research, Redeev Srl, Naples, ITA

**Keywords:** surprisal, confidence intervals, public health, testing, significance

## Abstract

Overconfidence in statistical results in medicine is fueled by improper practices and historical biases afflicting the concept of statistical significance. In particular, the dichotomization of significance (i.e., significant vs. not significant), blending of Fisherian and Neyman-Pearson approaches, magnitude and nullification fallacies, and other fundamental misunderstandings distort the purpose of statistical investigations entirely, impacting their ability to inform public health decisions or other fields of science in general. For these reasons, the international statistical community has attempted to propose various alternatives or different interpretative modes. However, as of today, such misuses still prevail. In this regard, the present paper discusses the use of multiple confidence (or, more aptly, compatibility) intervals to address these issues at their core. Additionally, an extension of the concept of confidence interval, called surprisal interval (S-interval), is proposed in the realm of statistical surprisal. The aforementioned is based on comparing the statistical surprise to an easily interpretable phenomenon, such as obtaining S consecutive heads when flipping a fair coin. This allows for a complete departure from the notions of statistical significance and confidence, which carry with them longstanding misconceptions.

## Editorial

**Figure 1:**
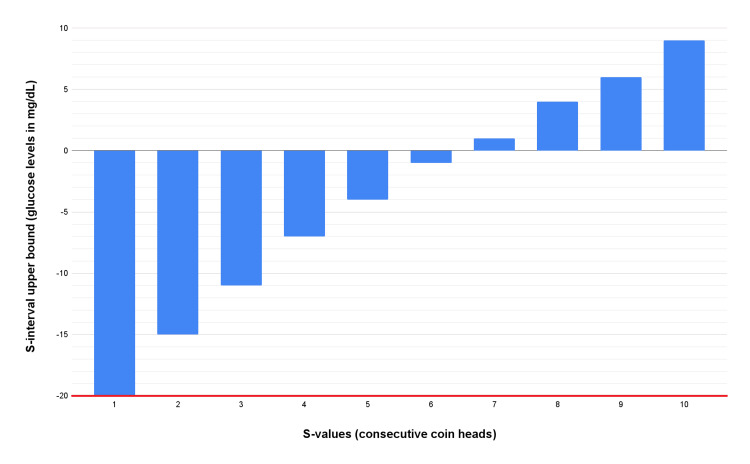
S-intervals (blood glucose levels in mg/dL) as a function of the respective S-values (statistical surprise compared to the number of consecutive heads when flipping a fair coin) according to the one-tailed Welch t-test. Red line = observed experimental result.

For decades, the scientific community has been plagued by dangerous misuses and abuses of statistical significance [1]. Especially in the field of public health, these errors can lead to severe consequences, such as the approval or endorsement by certain members of the scientific community for practices that have weak supporting evidence. In a time when information overload is a global issue, even within the mere research field, it is imperative that scientific standards become even more rigorous. However, the historical efforts of leading figures and organizations in the international statistical field have not been sufficient to address such longstanding problems to date, although they have been essential in limiting them. Specifically, the tendency to classify results as non-significant (e.g., P≥.05) and significant (e.g., P<.05) is mathematically incorrect and absurd, given that i) it improperly blends two mathematically incompatible approaches, namely Fisher's (which allows for the assessment of individual studies, most useful in the medical field) and Neyman-Pearson's (which assesses groups of studies and never individual ones), ii) as demonstrated by McShane et al., two results with very distant P-values (i.e., P=.005 and P=.194) can be extremely compatible with each other according to a certain test (i.e., P=.289), iii) statistical testing cannot prove or disprove a specific target hypothesis but can, at most, show the compatibility of such a hypothesis with experimental data through a specific statistical test, iv) statistical testing is mathematically disconnected from the scientific phenomenon, as it assumes that there is no other phenomenon outside chance (it is up to the researcher to interpret the numerical outcome considering the whole scientific scenario) [2, 3]. In addition, the magnitude fallacy (i.e., the inability to distinguish between statistical and practical relevance) and nullification fallacy (i.e., an obsession with the null hypothesis over other relevant hypotheses) consistently distort the vast majority of scientific analyses, as they have roots tied to the teaching of statistics in universities [1, 4]. In order to address these issues, this paper proposes an enrichment and expansion of some methodologies recently discussed by the statistical community. The first approach concerns traditional confidence intervals, which, in accordance with Greenland et al., should be termed compatibility intervals. Indeed, if and only if all assumptions of the statistical test have been properly verified, the P-value is an approximate index of the compatibility of experimental data with the target hypothesis according to the statistical test. P-values close to 1 indicate high compatibility, while P-values close to 0 indicate low compatibility. Thus, for example, assuming that all experimental and statistical procedures have been executed correctly, a 95% confidence interval of the form 95% CI = (a, b) states that all predictions of the target hypothesis between values a and b have approximately a statistical compatibility level with the experimental results greater than P=.05 (i.e., they all have P>.05). Let's consider a practical example: suppose that, after a certain treatment to reduce blood glucose levels, the test group recorded a mean of 120 mg/dL (SD 15 mg/dL) compared to a mean of 140 mg/dL (SD 20 mg/dL) in the placebo group. Both groups were of 10 patients. After verifying that our data were sufficiently compatible with the assumptions of the test (a vital aspect to which the researcher must give utmost importance, although, in this case, it goes beyond the scope of this paper), we apply a one-tailed Welch t-test to assess the situation statistically. Nonetheless, instead of solely assessing the null hypothesis, we evaluate degrees of compatibility that interest us. For instance, we calculate the following compatibility intervals: 99%, 97%, 95%, and 90% (that is, we seek all predictions that, in the aforementioned ways, result in P-values greater than .01, .03, .05, and .10 respectively). To present these results, we adopt a new notation that is very convenient to read, namely 99|97|95|90% CI = (-∞, 0|-∞, -4|-∞, -6|-∞, -9). This notation provides various pieces of information: e.g., assuming a one-sided target hypothesis of a difference h between h≥-4 and h≥-6, we would obtain a P-value ranging between .03 and .05. The observed experimental difference between the two groups (120-140 mg/dL = -20 mg/dL) has a compatibility of above .01 compared to the one-sided null hypothesis (h≥0). Additionally, we can observe how compatibility changes based on various hypothetical differences. In this case, we notice that we should consider the hypothesis of a minimum difference of -9 mg/dL to achieve P=.10, i.e., to achieve a moderate consistency with our result of -20 mg/dL. Therefore, we can reasonably deduce that the test shows good stability for our data. Nevertheless, even if we are satisfied at a statistical level, can we conclude the effectiveness of the treatment? No. In fact, we have not found evidence supporting the treatment's effectiveness but rather evidence compatible with it. There could be other equally plausible hypotheses for this result (e.g., an error in the random sampling procedure, a statistical coincidence or anomaly, etc.). Therefore, not only would further statistical confirmation be necessary, but there should be reasons of a different nature (e.g., clinical, biochemical, etc.) supporting the hypothesis of effectiveness. Furthermore, we have not assessed the cost-risk-benefit ratios, which are essential for making a public health decision (e.g., is the therapy invasive? Do the benefits justify the treatment?). However, compatibility intervals can create confusion since the amount of information in different P-value intervals is not constant (e.g., the difference between P_1_=.01 and P_2_=.05 contains much more information than the difference between P_3_=.95 and P_4_=.99 even if both ΔP=.04). This can pose problems with the interpretation of multiple compatibility intervals. In this regard, we introduce the concept of S-value [5]. Suppose we calculate the probability of obtaining S consecutive successes (heads) by flipping a fair (unbiased) coin. Since the probability of success in a single toss is P=1/2, for S flips, we have P=(1/2)^S^. It follows that S=-log_2_P (i.e., the base-2 logarithm of P). The main advantage is the comparison of the surprise of a statistical event to an everyday event that we have a very intuitive perception of. This way, it is easy to observe that P_1_=.01 implies S_1_=6.6, P_2_=.05 implies S_2_=4.3, P_3_=.95 implies S_3_=0.07, and P_4_=.99 implies S_4_=0.01. Thus, the difference between P_2_ and P_1_ is ΔS_1,2_=6.6-4.3=2.3, while the difference between P_4_ and P_3_ is ΔS3,4=0.07-0.01=0.06. The purpose and role of S-values are also to model the implicit uncertainty in each statistical analysis. In fact, obtaining exactly 6.6 consecutive heads is not possible, which is why, in decision logic, it is necessary to consider such a result as "a little more than 6 consecutive heads" or "a little less than 7 consecutive heads" or "between 6 and 7 consecutive heads." The relationship between S-values and compatibility intervals is not entirely clear, as it still has to go through the P-value. For this reason, the concept of S-interval has been introduced. In particular, assuming that all background assumptions have been verified, the S-interval contains all and only the target assumption predictions that are approximately less surprising than S consecutive heads - by flipping a fair coin - compared to the experimental result according to the statistical test. For example, a 4-I, under the conditions mentioned above, contains all the target assumption predictions less surprising than 4 consecutive heads. Calculating an S-I is straightforward; for instance, S=4 implies P=(0.5)^4^=.063, i.e., a compatibility interval of 100(1-P)%=100(1-0.063)%=93.7%. Resuming our example, we show the following S-intervals: 7|5|4|3-I = (-∞, 1|-∞, -4|-∞, -7|-∞, -11). We can thus see that, in order to achieve a hypothesis as surprising as 3 consecutive heads (i.e., moderately compatible with our result of -20 mg/dL), we need to consider a minimum supposed difference of -11 mg/dL. We can also observe that a non-negligible minimum hypothetical effect of -7 remains as surprising as obtaining 4 consecutive heads compared to our result of -20. The variation of the effect size as a function of statistical surprise (and vice versa) can also be graphically represented in order to be better evaluated (Figure 1). In general, it is observed that clinically small changes in effect size correspond to large changes in statistical surprise.

Hoping that these examples have clarified the potentials and limitations of the statistical approach in public health, the author of this manuscript invites the scientific community to consider such approaches for conducting statistically informative investigations at a scientific level. The methodologies presented here draw on previous literature, although the concepts of S-intervals and the proposed convention for reporting multiple intervals are, to the best of the author's knowledge, new.

## References

[1] Wasserstein RL, Lazar NA (2016). The ASA’s statement on p-values: context, process, and purpose. Am Stat.

[2] McShane BB, Bradlow ET, Lynch JG, Meyer RJ (2023). EXPRESS: “Statistical Significance” and statistical reporting: moving beyond binary. J Mark.

[3] Greenland S, Senn SJ, Rothman KJ, Carlin JB, Poole C, Goodman SN, Altman DG (2016). Statistical tests, P values, confidence intervals, and power: a guide to misinterpretations. Eur J Epidemiol.

[4] Kühberger A, Fritz A, Lermer E, Scherndl T (2015). The significance fallacy in inferential statistics. BMC Res Notes.

[5] Greenland S, Mansournia MA, Joffe M (2022). To curb research misreporting, replace significance and confidence by compatibility: a preventive medicine golden jubilee article. Prev Med.

