# Correction: Multiple Confidence Intervals and Surprisal Intervals to Avoid Significance Fallacy

**DOI:** 10.7759/cureus.c161

**Published:** 2024-02-14

**Authors:** Alessandro Rovetta

**Affiliations:** 1 Research and Disclosure Division, R&C Research, Bovezzo (BS), ITA; 2 Technological and Scientific Research, Redeev Srl, Naples, ITA

This article has been corrected by the journal at the request of the author. Due to an error in the calculation of the example statistical test (Welch t-test), the compatibility and surprisal intervals previously presented have been replaced with the correct ones. Specifically, the compatibility intervals had been mistakenly replaced with those commonly defined as acceptability ranges.

- Wrong (old): 99|97|95% CI = (-20, +∞|-16, +∞|-14, +∞)
- Fixed: 99|97|95|90% CI = (-∞, 0|-∞, -4|-∞, -6|-∞, -9)

- Wrong (old): 7|5|4-I = (-21, +∞|-16,+∞|-13, +∞)
- Fixed: 7|5|4|3-I = (-∞, 1|-∞, -4|-∞, -7|-∞, -11)

The interpretation of the outcomes and the associated Figure 1 have also been adjusted accordingly. It is now specified that the rapid variation in compatibility and surprise would be indicative of instability in a neighborhood of the null hypothesis.

It is important to note that the validity of the concepts of multiple compatibility intervals and surprisal intervals remains unchanged, as only the results of the example test and their interpretation have been modified. Likewise, all arguments concerning P-values and S-values also remain unchanged.

